# Effects of Chinese Cooking Methods on the Content and Speciation of Selenium in Selenium Bio-Fortified Cereals and Soybeans

**DOI:** 10.3390/nu10030317

**Published:** 2018-03-07

**Authors:** Xiaoqi Lu, Zisen He, Zhiqing Lin, Yuanyuan Zhu, Linxi Yuan, Ying Liu, Xuebin Yin

**Affiliations:** 1 School of Earth and Space Sciences, University of Science and Technology of China, Hefei 230026, China; lusokey@163.com (X.L.); hezisen@mail.ustc.edu.cn (Z.H.); 2 Department of Environmental Sciences, Southern Illinois University, Edwardsville, IL 62026-1099, USA; zhlin@siue.edu; 3 Suzhou Setek Co., Ltd., Suzhou 215123, China; zhuyuanyuan718@126.com (Y.Z.); yuanlinxi001@gmail.com (L.Y.); liuin624@163.com (Y.L.)

**Keywords:** cereals, soybean, selenium speciation, cooking, biofortification

## Abstract

Cereals and soybeans are the main food sources for the majority of Chinese. This study evaluated the effects of four common cooking methods including steaming, boiling, frying, and milking on selenium (Se) content and speciation in seven selenium bio-fortified cereals and soybeans samples. The Se concentrations in the selected samples ranged from 0.91 to 110.8 mg/kg and selenomethionine (SeMet) was detected to be the main Se species. Total Se loss was less than 8.1% during the processes of cooking except milking, while 49.1% of the total Se was lost in milking soybean for soy milk due to high level of Se in residuals. It was estimated that about 13.5, 24.0, 3.1, and 46.9% of SeMet were lost during the processes of steaming, boiling, frying, and milking, respectively. Meanwhile, selenocystine (SeCys_2_) and methylselenocysteine (SeMeCys) were lost completely from the boiled cereals. Hence, steaming and frying were recommended to cook Se-biofortified cereals in order to minimize the loss of Se.

## 1. Introduction

Selenium (Se) is an essential micronutrient both for humans and animals. It forms an important component of glutathione peroxidase as the well-known antioxidant combating oxidative damage at cellular level [1]. In addition, Se plays an important role in catalyzing the production of active thyroid hormone [2,3], and is required for improving human immunity and sperm mobility [4,5]. Meanwhile, Se deficiency has been associated with the endemic cardiomyopathy called Keshan disease and a type of osteoarthritis (Kashin–Beck disease) reported primarily in northeast China or in low soil-Se regions [6,7]. Epidemiological survey indicated that Se deficiency was positively correlated with the incidence of cancer [8].

According to World Health Organization (WHO), China is one of the 40 countries assigned as a low-Se or Se deficient area [9]. Soil-Se deficiency was observed in China from northeast to southwest, including 22 provinces and districts. Moreover, approximately 70 million people suffered from potential adverse health impacts of Se deficiency. In the 1990s, the dietary Se-intake of Chinese adult women was only 35.1 μg/day [10], which is much less than the Se recommended daily allowance (RDA) of 60 μg/day. Based on previous studies, the Suzhou metropolitan region was moderately deficient in Se [11], and the estimated daily Se dietary intake was 43.9 μg/day. Additionally, it was also speculated that people living in suburban areas consumed more cereals which usually contain less Se. Thus, the daily Se dietary intake in rural areas of Suzhou would be even less than 43.9 μg/day. Meanwhile, the measurement of Se content in 408 human hair samples collected from Chinese inhabitants across northeast to southeast regions indicated that hair Se contents decreased by 24–46% compared with past residents in the same geographic region, which associates with the decrease of grain consumption as well as the lower Se content in the staple food rice [12].

Diet is the main source of Se intake for most people in developing countries. The Se accumulated in regular food is primarily in the organic form which can be easily absorbed by human body [13,14]. However, excess amounts of Se intake could lead to toxic effects depending on the species, oxidation state, and concentration of Se [15,16,17]. Due to the instability and volatility of Se compounds, Se could be lost during various cooking processes. Hence, the relevant studies have been conducted to determine the effects of cooking methods on the Se content or loss in cooked food [18,19,20]. The effect of cooking on the Se content in a variety of foodstuffs typically found in the American diet was studied [21], indicating that there was little or no significant loss of Se in the process of broiling meats, baking seafood, frying eggs, and boiling cereals, while there is a 7–23% loss of Se that occurs when drying and heating cereals. Boiling two vegetables (mushrooms and asparagus) that contained relatively high amounts of Se even led to 29% and 44% losses of Se, respectively. However, no significant differences of Se contents were found from raw and cooked sea foods consumed in Portugal [22]. Compared with western countries, China generally consumes more cereals and vegetables than meats. Considering the different diet structure and cooking methods, Chinese cooking methods may have different effects on Se content in cooked foods. However, as a country with low Se or Se deficiency, there have been few published studies on effects of cooking processes on the Se loss from processing foods. Furthermore, it is possible that high cooking temperature may alternate the speciation or chemical composition of Se in cooked foods. Since the Se speciation is an important factor of food Se bio-accessibility and bioavailability [23], it is significantly important to investigate how the Se speciation changes during the cooking process. Various detection methods have been developed to detect the selenium speciation. Separation of selenium species in food samples is generally carried out by high-performance liquid chromatography (HPLC). The most widely used liquid chromatographic modes are reversed phase ion pair [24] and ion-exchange chromatography [25]. Also, there are several detection techniques which could be utilized for selenium determination, such as inductively coupled plasma mass spectrometry (ICP-MS) [26], atomic absorption spectrometry (AAS) [27], and atomic fluorescence spectrometry (AFS) [28,29]. AFS is a very sensitive, selective, and low-cost method for the determination of selenium, and was chosen for selenium species detection in this study. In the present study, four representative cooking methods widely used in China were chosen to determine how the cooking methods affected the Se content and speciation in Se bio-fortified cereals and soybeans.

## 2. Materials and Methods

### 2.1. Reagents and Standards

Deionized water (Millipore system) was used in this study for chemical preparation. All chemicals used (e.g., Tris, HCl, H_2_O_2_, HCOOH, NaOH and (NH_4_)_2_HPO_4_) were analytical grade or above, provided by Sinopharm Chemical Reagent (Shanghai, China). A 1000 mg/L Se stock standard solution was purchased from Merck Millipore (Darmstadt, Germany). Besides, Protease K (30 U/mg), Protease XIV (5.5 U/mg), selenomethionine (SeMet, >98%), selenocystine (SeCys_2_, >98%), and Se-methylselenocysteine (SeMeSeCys, >98%) were obtained from Sigma-Aldrich (St. Louis, MO, USA).

### 2.2. Sample

Three different Se-enriched cereals (including one rice, one wheat and two corn samples) and soybeans (including three soybean samples) were selected for this study. These Se-biofortified samples were produced and provided by Suzhou Setek Co., Ltd. (Suzhou, China). The grain samples were oven-dried at 45 °C for 48 h, which were subsequently stored at room temperature until cooking or conducting chemical analysis.

### 2.3. Cooking Methods

Four cooking methods including steaming, boiling, frying, and milking (only for soybeans) were employed in the current work. The detailed processes of these four cooking methods were described in Table 1. Steamed foods were especially popular in China, such as steamed rice, steamed bread, and steamed fish/seafoods. For Chinese people, boiling is also a main cooking method of cereals for breakfast and dinner. Frying and milking cooking processes are also very common cooking methods for breakfast, such as preparing fried Chinese doughnut (or Youtiao) and soy milk.

### 2.4. Sample Preparation and Determination of Total Selenium

The procedure for determination of total Se was described in the previous study [11]. In brief, 0.2 g of ground/powdered samples was added into a 50-mL conical flask followed by addition of 2 mL concentrated HClO_4_ and 8 mL concentrated HNO_3_ into each flask at room temperature for overnight. Subsequently, the solution was heated on an electrical hot plate for 1 h at 100 °C, 2 h at 120 °C, 1 h at 180 °C, and then kept it at 210 °C until the formation of white fume appeared formed. 5 mL concentrated HCl was added to reduce Se^VI^ to Se^IV^ for at least 4 h. Then, each solution was diluted using double-deionized water to the final total volume of 25 mL and the detection of Se was performed by hydride generation atomic fluorescence spectrometry (HG-AFS 9230) (Beijing Titan Instrument Co., Beijing, China).

### 2.5. Sample Preparation and Determination of Selenium Speciation

0.1 g of powdered sample was transferred to a 10-mL plastic tube, and 5 mL Tris-HCl (100 mmol/L, pH = 7.5) was added into the plastic tube. The plastic tube was then homogenized by ultrasound for 30 min. Subsequently, 0.4 mg of the enzyme Protease K dissolved in 0.4 mL deionized water was added into the tube and the mixture was incubated at 50 °C on a shaker at 250 rpm for 24 h. Afterwards, 0.2 mg of the enzyme Protease XIV was added to the mixture, which was subsequently incubated on a shaker at 250 rpm and 37 °C for another 18 h. After the extraction process, the mixture solution was centrifuged at 10,000 rpm for 30 min (Sigma 3K15). The supernatant was collected and filtered through a 0.22 µm hydrophilic filter to collect the solutions, which were stored at 4 °C until Se speciation analysis.

**Figure 1:**
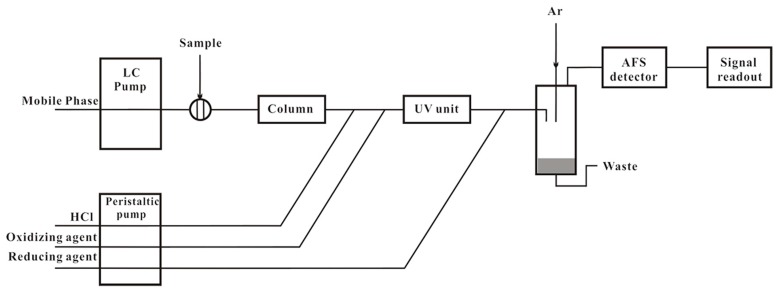
Scheme of the liquid chromatogram hydride generation atomic fluorescence spectrometry (LC-HG-AFS) system.

**Figure 2:**
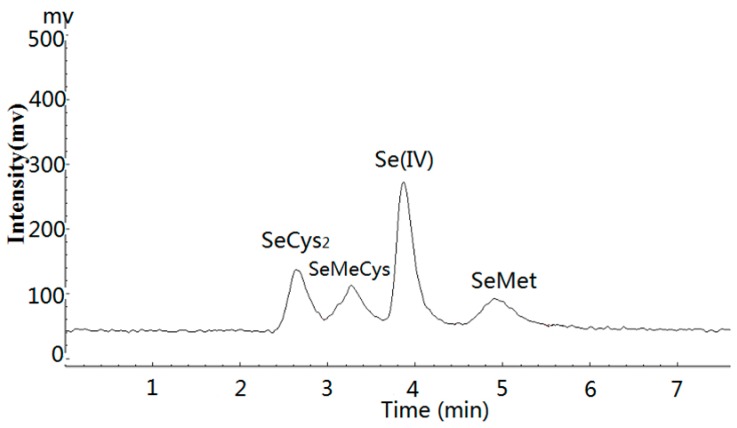
Chromatogram of the four Se standard mixture including SeCys_2_, SeMeCys, Se(IV), and SeMet. Concentrations of the Se standards were 100 µg/L.

HG-AFS was coupled as a detector to a liquid chromatographic system (LC-20AB, SHIMADZU) for Se speciation analysis. A Hamilton PRP X-100 anion exchange column was used as the stationary phase. The analyses of extracts were performed utilizing the anion exchange column. The scheme of the system of liquid chromatogram hydride generation atomic fluorescence spectrometry (LC-HG-AFS) was displayed in Figure 1 and liquid chromatogram (LC) conditions were presented in Table 2. The eluent from the column was mixed with 10% HCl (*v*/*v*, flow rate 1 mL/min) and oxidizing agent solution (0.8% H_2_O_2_ in 3.5 g/L NaOH, flow rate 1 mL/min), which was subsequently passed through the ultraviolet unit. H_2_Se generated by reaction with reducing agent (1.2% NaBH_4_ in 3.5 g/L NaOH) was transported by carrier gas (Argon, 300 mL/min) through a gas-liquid separator into AFS. A chromatogram for the four Se standard mixture solutions containing 100 µg/L of each selenium compound was shown in Figure 2. It should be pointed out that SeCys_2_ eluted at the void volume of the separation and other unretained selenium species would coelute, therefore the SeCys_2_ peak represented unretained selenium species in cereals and soybeans in general.

## 3. Results and Discussion

### 3.1. Se Concentrations of Bio-Fortified Cereals and Soybeans

Concentrations of Se in seven cereal and soybean samples were included in Table 3. Standard reference material GBW 07602-GSV-1 (shrub leaves) was used for food samples. The recovery of the standard reference materials ranged from 85.5% to 117.8% and the relative standard deviation (RSD) of reference materials was calculated as 0.76%. The method detection limits for Se was evaluated on the basis of the standard deviation (SD) of the signals measured (15 times) for the blank solution, and the detection limit of HG-AFS method for samples was 0.08 µg/kg. The Se content was ranged from 0.91 to 110.8 mg/kg, while Se level of non-biofortified cereals and soybeans samples collected from the same sampling place ranged from 0.007–0.023 mg/kg. Concentrations of Se in the Se-biofortified cereals and soybeans were also significantly higher than those of common cereals and soybeans that were determined in other previous studies. For example, concentrations of Se in cereals in northwestern Spain, southeastern Spain, and the Slovak Republic were 0.015–0.09, 0.002–0.078, and 0.012–0.057 mg/kg, respectively [30,31,32], while soybean Se concentrations in southeastern Spain was 0.018–0.269 mg/kg [31]. Moreover, Se levels of bio-fortified cereals and soybeans in the current work were higher than the Se levels of cereals (0.13–1.93 mg/kg) and soybeans (0.46–1.37 mg/kg) collected from Enshi, a high Se region in Hubei, China, where human selenosis had been observed between 1958 and 1963 [33]. Therefore, it can be seen that Se biofortification was an effective way to increase the Se concentrations in cereals and soybeans in soils with low Se background levels.

The dietary Se intake from cereal-based diet was generally insufficient due to the relatively inadequate amount of Se in cereals compared with meat, poultry, and fish. The dietary Se intake in Suzhou was revealed to be 43.9 µg/day by a previous investigation [11], which partly resulted from primary cereal-based diet. It is clear that the dietary Se intake by residents in Suzhou was still below the recommended Se dietary allowance of 60 µg/day for adult proposed by Chinese government. Therefore, Se-biofortification for cereals was essential for habitants living in cereal-based diet areas with low Se geological background. The use of Se-biofortification bore the fruits of success in Finland. Briefly, due to the low Se content in soil, the Finnish government made an official decision in 1984 to supplement the agricultural soil fertilizers with sodium selenate. Before the soil Se amendment program, Se concentrations in Finland domestic cereals were less than 0.01 mg/kg, which was effectively increased by the application of Se-biofortification. The increase of soil Se content significantly enhanced the Se accumulation in crops. As a result, the average daily dietary Se intake by Finnish people increased to about 110 µg/day, satisfying the requirements of Se for human health [34].

### 3.2. Se Speciation of Bio-Fortified Cereals and Soybeans

Se speciation of five samples with high Se level was shown in Table 4. A standard mixture solution (20 µg/L) of SeCys_2_, SeMeCys, SeMet, and Se (IV) was measured 11 consecutive times, with the RSD of 2.8, 3.3, 2.9, and 2.0%, respectively. The instrument detection limits for SeCys_2_, SeMeCys, SeMet and Se (IV) were 2.3, 4.9, 7.4, and 2.0 µg/L (100 µL injection, three-times the baseline noise), respectively. The recovery rates for SeCys_2_, SeMeCys, SeMet, and Se (IV) were 89.4–90.8%, 80.6–95.8%, 85.2–92.7%, and 91.6–97.6%, respectively. Due to the limitation of the detector’s sensitivity of the detector, Se speciation of rice and soybean C was not detected. Four dominant Se species were identified in the studied samples according to the retention times of available standards. The recovery of the sum of identified species to the total Se varied from 47.1% to 89.0%. Consistently with other previous studies [35,36], the predominant extractable Se species in the cereal and soybean samples was SeMet accounted for 44.2% to 80.4% of the total Se, followed by SeCys_2_ and SeMeCys which accounted for 1.6% to 6.9% and 0.3% to 4.2%, respectively. No selenite was detected in wheat and two corn samples, while little selenite was detected in two soybean samples, possibly due to the rapid conversion of selenite to organic forms of Se, such as SeMet [37].

### 3.3. Effects of Cooking on Se Content

**Figure 3:**
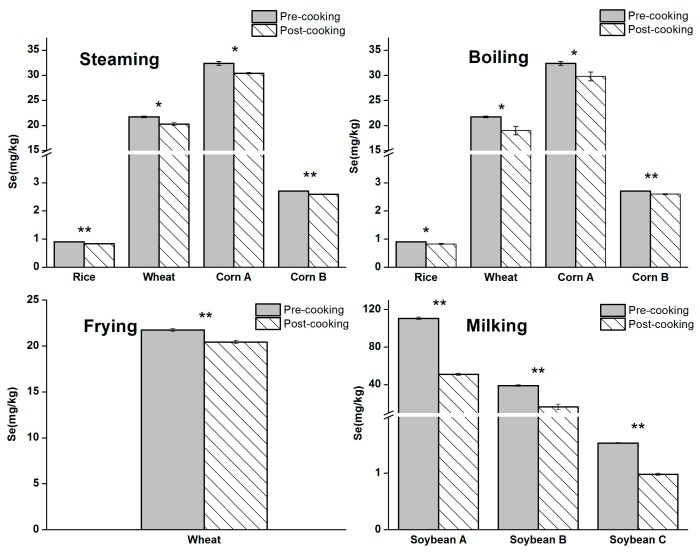
Selenium loss from the cereals and soybeans in the processes of the four cooking methods (*n* = 3). * means *p* < 0.01, ** means *p* < 0.001 (two sample t test on normally distributed data, Origin Pro 8, (Origin Lab, Northampton, MA, USA.)).

The effects of four cooking methods on Se content were shown in Figure 3. Se content in cooked cereals was reduced by 4.2% to 7.8% of the total Se accumulated in the cereals, while cereals were streamed at 100 °C for 30 min. It was estimated that boiling caused 4% to 12.2% Se loss, while frying caused 5.6% of Se loss. Another study demonstrated that the common cooking procedure (boiling) in China prior to consumption did not change total Se levels in the rice [38]. This may be due to the difference on boiling parameters in two studies. In the referenced study, rice was boiled at rice:water ratios (1:3) until all of the water is absorbed into the rice, while cereals were boiled at cereal:water ratios (1:10) and plenty of water was left after boiling in this study. In general, the Se loss from the cereals during steaming, boiling and frying cooking processes in the current work was less than 13%. Specifically, compared with steaming, boiling, and frying, milking soybean for soy milk resulted in higher Se loss, which was 53.8, 57.5, and 36% in soybeans A, B, and C, respectively.

As shown in Figure 3, all cooking methods could cause Se loss. Se loss of cereals caused by steaming, boiling, and frying was 6.1, 8.1, and 5.6%, on average. Although the majority of selenium was retained in the cooked cereals, milking soybean for soy milk caused a higher percentage loss of Se, which was as high as 49.1% on average. The lost Se can be converted into the aqueous (or oil) phase, or be absorbed on the walls of the utensils, or escape in the gaseous phase [18]. The frying oil and boiling water was also analyzed for Se after the processes of frying and boiling. The results showed that 0.4% and 2.3% to 9.8% of Se was presented in the frying oil and boiling water after cooking. A large amount of residue was produced in the process of milking. Se concentration of residue produced in the process of milking for soybeans A, B, and C was 55.2, 20.5, and 0.53 mg/kg (calculated by the pre-cooked dry sample matrix weight), respectively. The residue accounted for majority of Se loss in the process of milking, which was 92.5, 91.7, and 94.6% for soybean A, B, and C, respectively.

### 3.4. Effects of Cooking on Se Speciation

All of the Se species in the Se bio-fortified cereals and soybeans were lost to some extent during the processes of four cooking methods (Table 4). SeMet was the dominant chemical form of Se in the cereals and soybeans, as well as in the cooked cereals and soybeans. Besides, SeMet loss in the process of steaming, boiling, frying, and milking were less than 13.5, 24.0, 3.1, and 46.9%, respectively. Thiry et al. reported that the degrees of bioavailability of pure individual Se species was selenite (12%) < selenate (33%) < SeMeCys (46%) < SeMet (56%) [23]. Due to the capacity of SeMet to integrate into proteins, Se can thus be progressively released into the organism during the regular turnover of proteins and continuously meet the body requirements of Se. Moreover, SeMet was demonstrated to be less toxic than inorganic species [39]. Therefore, SeMet in the Se-enriched cereals should be considered as good sources for long-term Se supplementation.

Compared with steaming and frying, a higher percentage loss of SeMet occurred in the process of boiling. Additionally, SeCys_2_ and SeMeCys were lost completely in the boiled cereals. More Se of these species was lost in the process of boiling, probably because these Se species escaped to liquid water more easily than water vapor or oil, especially SeCys_2_ and SeMeCys. In comparison with steaming, higher percentage loss of SeCys_2_ occurred in the process of frying, possibly due to the decreased stability of SeCys_2_ at high temperature. During the process of milking, soybean lost the highest percentage of Se species due to the high Se content in the residue. In consideration of the remaining Se species in cooked foods after cooking with different methods, steaming and frying were recommended to cook Se bio-fortified cereals.

## 4. Conclusions

In conclusion, this study demonstrated that SeMet was the main Se species in Se bio-fortified cereals and soybeans. Se content loss in the process of steaming, boiling, and frying was less than 8.1%, while 49.1% of Se content on average was lost in milking soybean due to high Se content in the soybean residue. SeMet losses in the process of steaming, boiling, frying, and milking were 13.5, 24.0, 3.1, and 46.9%, respectively, while SeCys_2_ and SeMeCys were lost completely in the boiled cereals. In order to reduce Se loss, steaming and frying were recommended to cook Se bio-fortified cereals.

## Figures and Tables

**Table 1:** Description of processes for cooking methods

Cooking Method	Process Description
Steaming	100 g cereals in a container were placed in a steamer with boiling water vapor and heated for 30 min.
Boiling	100 g cereals were put into a container with 1000 g boiling water and heated for 30 min.
Frying	Cereals were smashed to powder over a 0.425 mm sieve. 100 g cereals powder and 50 g cold water were mixed into dough. The dough was evenly divided into 30 equal parts and fried in soybean oil at a temperature of 200 °C for 5 min.
Milking	Soybean was smashed to powder over a 0.425 mm sieve. 100 g soybean powder were boiled in 2000 mL water at the temperature of 100 °C for 30 min. The liquid called as soy milk produced by filtering the mixture by using a 0.250 mm sieve.

**Table 2:** Optimized instrumental parameters for liquid chromatogram (LC).

LC Parameters	
Column	Hamilton PRP X-100 (250 mm × 4.1 mm × 10 μm)
Mobile phase	40 mM NH_4_H_2_PO_4_ (pH 6.0)
Flow rate	1 mL/min
Injection volume	100 μL

**Table 3:** Concentrations of selenium in seven cereals or soybean samples obtained from Se biofortified agricultural products (*n* = 3)

Samples	Mean ± SD * (mg/kg)
Rice	0.91 ± 0.01
Wheat	21.7 ± 0.25
Corn A	32.4 ± 0.38
Corn B	2.71 ± 0.01
Soybean A	110.8 ± 12.8
Soybean B	39.1 ± 1.0
Soybean C	1.54 ± 0.01

* SD is standard deviation.

**Table 4:** Selenium species concentration of pre-cooking and cooked cereals and soybeans.

Samples	Cooking	SeCys_2_ (Se, mg/kg)	SeMeCys (Se, mg/kg)	Selenite (Se, mg/kg)	SeMet (Se, mg/kg)
Wheat	Pre-cooking	0.67 ± 0.13	0.14 ± 0.08	ND *	9.6 ± 0.1
	Steaming	0.57 ± 0.12	0.09 ± 0.05	ND	8.3 ± 0.2
	Boiling	ND	ND	ND	7.3 ± 0.1
	Frying	0.29 ± 0.03	0.10 ± 0.03	ND	9.3 ± 0.1
Corn A	Pre-cooking	0.72 ± 0.22	0.21 ± 0.05	ND	20.5 ± 2.5
	Steaming	0.43 ± 0.03	0.18 ± 0.02	ND	19.6 ± 0.2
	Boiling	ND	ND	ND	17.7 ± 1.1
Corn B	Pre-cooking	0.12 ± 0.02	0.11 ± 0.03	ND	2.2 ± 0.4
	Steaming	0.08 ± 0.02	0.08 ± 0.01	ND	2.0 ± 0.1
	Boiling	ND	ND	ND	1.7 ± 0.1
Soybean A	Pre-cooking	7.7 ± 0.9	1.4 ± 0.1	3.7 ± 0.9	55.7 ± 0.2
	Milking	3.2 ± 0.5	0.79 ± 0.27	ND	29.6 ± 1.1
Soybean B	Pre-cooking	0.64 ± 0.13	1.1 ± 0.1	0.35 ± 0.04	25.7 ± 3.5
	Milking	0.21 ± 0.03	0.29 ± 0.16	ND	16.6 ± 2.9

Results are expressed as mean value ± standard deviation (*n* = 3). * ND means no n-detectable. All the Se species concentrations of cooked cereals and soybeans were calculated by the pre-cooked dry sample matrix weight.
